# Integrated Management of Skin NTDs—Lessons Learned from Existing Practice and Field Research

**DOI:** 10.3390/tropicalmed3040120

**Published:** 2018-11-14

**Authors:** Rie R. Yotsu

**Affiliations:** 1 School of Tropical Medicine and Global Health, Nagasaki University, Nagasaki 852-8102, Japan; ryotsu@nagasaki-u.ac.jp; 2 Department of Dermatology, National Center for Global Health and Medicine, Tokyo 162-8655, Japan

**Keywords:** case management, integration, mass drug administration, neglected tropical diseases, skin infections, skin NTDs, surveillance, training, tropical skin diseases

## Abstract

Integration of neglected tropical diseases (NTDs) into the public health agenda has been a priority in global health for the last decade. Because a number of these diseases share not only the geographical distribution, but also a common feature which is skin involvement, bringing together a sub-group of ‘skin NTDs’ is one way forward to promote further integration among NTDs. With these diseases, which include leprosy, Buruli ulcer, yaws, mycetoma, lymphatic filariasis, and leishmaniasis, patients may be left with life-long deformities and disabilities when diagnosis and treatment are delayed. Stigma is another serious consequence of skin NTDs as it places a large barrier on the economic activities and social life of a patient. As a result, this creates a vicious cycle and obstructs a key goal of society, the elimination of poverty. Enhancement in surveillance systems as well as the further development of diagnostic methods, improvement in treatment and management, and identification of preventative measures for skin NTDs are therefore urgently needed. This article summarizes the existing practices and field research on skin NTDs and identifies potential synergies that could be achieved by adopting this integrated approach.

## 1. Introduction

Neglected tropical diseases (NTDs) are a group of infectious diseases that prevail in tropical and sub-tropical regions, affecting impoverished populations living in conditions of poor sanitation and in close contact with infectious vectors and livestock; such communities also have very limited access to adequate healthcare [1]. It is estimated that over one billion people in 149 countries are affected [2]. In May 2013, the World Health Assembly (WHA) adopted resolution WHA66.12, which calls on Member States to intensify and integrate measures against these NTDs to effectively and efficiently enhance the health and social well-being of the affected populations [2].

Successful integration of diagnostic and therapeutic interventions has happened for some NTDs, particularly among those that can be managed at community level by mass drug administration (MDA). This set of diseases forms the basis of a sub-group sometimes known as preventative chemotherapy and transmission control (PCT) NTDs, which includes such diseases as cysticercosis, foodborne trematode infections, lymphatic filariasis, onchocerciasis, schistosomiasis and soil-transmitted helminthiasis. Dracunculiasis (guinea-worm disease) is reaching close to eradication through transmission control; however, the recent transmission seen in dogs is hindering the last drive in Chad, which may show further spread [3]. In contrast, there are other NTDs where early diagnosis and management on an individual basis may be the only workable therapeutic measure, at least at present, and these are categorized as innovative and intensified disease management (IDM) NTDs. As IDM NTDs need considerable resources, including personnel with skills and financial support, and these measures do not produce a dramatic and immediately visible impact, which may lead to lower investment in research and development, the control of this set of diseases is lagging behind the PCT-NTDs [2].

**Figure 1:**
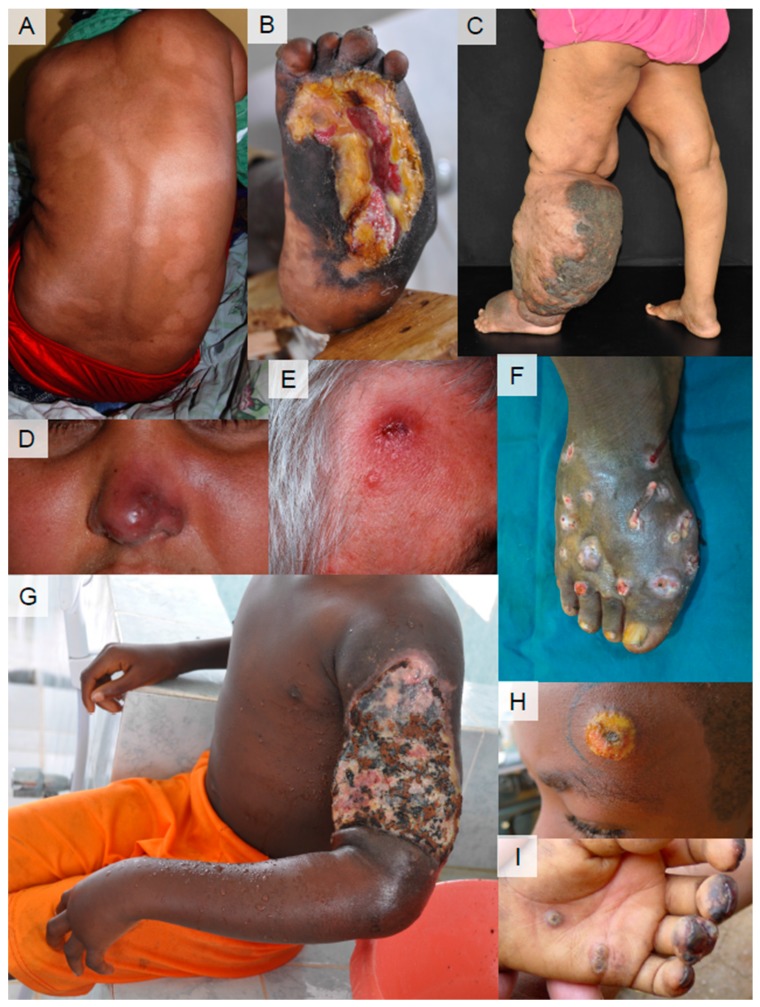
Clinical presentation of skin NTDs. (**A**) Leprosy (borderline tuberculoid leprosy). Ill-defined, multiple hypo-pigmented patches on the back. (**B**) Deformities of the feet and ulcer from peripheral neuropathy in leprosy. (**C**) Lymphatic filariasis. Unilateral lymphedema of the limb. (image: Saravu R. Narahari) (**D**) Mucocutaneous leishmaniasis. Redness and swelling of the nose. Inside: destruction of the nasal mucosa. Same patient as (**A**) (co-infection). (**E**) Cutaneous leishmaniasis. Infiltrated granulomatous lesion with central ulceration on the forehead. (**F**) Mycetoma. Multiple nodules with openings of draining sinuses discharging pus and blood. (image: Ahmed Fahal) (**G**) Buruli ulcer. Ulceration on the arm with extensive edema. Black and yellowish necrotic tissue on wound surface with some traditional remedies at first visit. (**H**) Yaws (primary yaws). Nodule with central ulceration with yellow crust on the forehead. (image: Kingsley Asiedu) (**I**) Tungiasis. Multiple small nodules with central black dot (body part of the adult flea) on the palm and on the finger tips.

Interestingly, a number of IDM-NTDs share a common feature, which is involvement of the skin, for example, in the forms of nodules, patches, edema, and ulceration (Figure 1). To assist in a breakthrough in the above-mentioned gap in the global health agenda, there is a move designed to improve integrated strategies to group these NTDs with skin manifestations under the banner of skin NTDs [4,5]. Among the skin NTDs are leprosy, Buruli ulcer, yaws, cutaneous and mucocutaneous leishmaniasis, chromoblastomycosis and mycetoma, and ectoparasites including scabies and tungiasis. Human African trypanosomiasis and post Kala-azar dermal leishmaniasis may present with skin signs, which although not the main symptom of the disease, may aid in early detection. Lymphatic filariasis and onchocerciasis are also skin NTDs, which are controlled by MDAs, but as control measures are rolled out across endemic areas there will be a residue of individual cases that have escaped detection or preemptive therapy with MDAs, whose recognition and treatment is essential in order to achieve effective control. Yaws and scabies are skin NTDs that are potentially controlled or eliminated through preventative chemotherapy, and the expansion of this approach on a larger scale is currently under study [6,7,8,9,10].

In addition, there are other skin diseases that are not formally recognized by WHO as NTDs that contribute a huge disease burden in impoverished populations. These include podoconiosis (a geochemical, non-filarial elephantiasis due to long-term contact with irritant red clay soil), fungal and bacterial skin infections, and tropical ulcers [11,12]. These diseases should also be considered as neglected. However, their management as public health problems may also benefit from this skin NTDs integrative approach.

Surveillance and early detection, MDA, and case and morbidity management are potential areas for integrative initiatives for skin NTDs. This paper is not a systematic review of all skin NTDs, but rather it summarizes the current state of knowledge and lessons learned from existing practice and field research to aid in effective project implementation for managing skin NTDs.

## 2. Active Surveillance

There are several factors that hinder early detection and treatment of skin NTDs. Skin NTDs are often painless or accompanied by limited discomfort—this feature prevents patients and families from presenting to healthcare facilities at an early stage [13,14,15]. Low awareness of the disease among the populations at the highest risk also adds to the delay in seeking help, and this is compounded by the consequences of stigma and discrimination often associated with these diseases [13,14,15,16]. Many patients tend to seek treatment preferentially with traditional healers rather than at health care centers, as there is easier access and lower cost [15,16,17]. This health seeking behavior is also linked to the common perception that the diseases may result from mystical causes (witchcraft and curses) [15,16,17]. 

In the light of current perceptions among health authorities and communities, and given the lack of mass population measures or field-friendly diagnostic tests, active surveillance is still the most effective measure for early detection and thereby, treatment for most skin NTDs in order to prevent patients from developing disabilities, disfigurement, and comorbidities. Early detection and treatment are also key to cutting further transmission of the diseases, for instance, in leprosy, scabies, and yaws, and this underlines the importance of active surveillance.

As skin diseases can be detected by visual examination—a unique feature in this set of diseases—that if used effectively, for instance by organizing community skin surveys or treatment initiatives, is one simple approach for detection of skin NTDs that would otherwise remain hidden. Outreach skin surveys have been conducted with successful results in the case of leprosy in different parts of the world, for example, in India, Pacific Islands, Malawi, Cameroon, and recently in Brazil [18,19,20,21,22,23,24,25]. The Brazilian project led by the Ministry of Health targeted more than nine million schoolchildren aged between 5–14 years for skin screening of leprosy coupled with de-worming (albendazole for soil-transmitted helminthiases) [25]. In Japan where leprosy is no longer prevalent, school skin surveys played a considerable role in achieving a decline in cases during the 1970s in Okinawa, the most southern island of Japan, which remained as the last pocket during the endemic era [26]. Consecutive surveys at 3–5 year intervals were behind this success.

As part of promoting integration of skin NTDs, in line with the current global approach, there have been newer studies. An observational study in Cameroon reviewed the different means of detection of a total of 815 cases of yaws identified during the three-year period between 2012 and 2015 in one endemic region [27]. They reported that these cases were reported through the synergistic effect of five approaches: passive yaws detection at local clinics after training of local staff on NTDs, small community-based NTD case detection activities, community-based yaws screening immediately following Buruli ulcer outreach programs, school-based screening, and house-to-house searches. Rapid cure of yaws increased the uptake of, and confidence in, the treatment of Buruli ulcers, and thus led to a “win-win” situation for the two diseases.

In Côte d’Ivoire, another West African country, school skin surveys in areas identified as co-endemic for leprosy, Buruli ulcer, and yaws are also in the process of implementation [12]. The project is sometimes coupled with other activities, such as de-worming, if the timing coincides, to achieve resource mobilization as well as increased acceptance by the community. In this project, the latter is also enhanced by targeting all skin diseases, a strategy that also prevents creating unnecessary feelings of stigma and discrimination against skin NTDs. Targeting all skin diseases therefore appears to be a way forward. However, a careful strategy needs to be prepared as there is usually a high prevalence of skin diseases in such communities where skin NTDs are prevalent—ranging from infectious to inflammatory. This may pose a practical challenge in project implementation, i.e., cost of medications and ensuring referral pathways. In communities in African countries where skin NTDs are likely to be endemic, prevalence of skin diseases can range from 26% to 80% [12,28,29,30,31,32,33].

*Key points in implementing integrated surveillance:*

1. Selection of the intervention area: try to collect past data and develop mapping methods to identify co-distribution of cases of skin NTDs
2. Training of local healthcare workers both on skin NTDs and common skin diseases
3. Treatment: develop protocols on how to manage the different diseases anticipated; be prepared to treat or to refer
4. Look for opportunities for integration with other community- or school-based activities, e.g., de-worming, vitamin A and micronutrient supplementation, onchocerciasis and/or lymphatic filariasis control, to gain a synergic effect
5. Plan for repeat rounds/follow-up activities, decide on the appropriate intervals.

## 3. Mass Drug Administration and Prophylaxis

Lymphatic filariasis and onchocerciasis are already operationally covered by active MDA programs. In addition, several other skin NTDs are thought to be controllable using MDAs, and field research is ongoing.

Yaws comes first on that list, and recent studies have shown that high coverage with MDA consisting of single-dose azithromycin may be effective in control of the disease. A study conducted in an island off Papua New Guinea (16,092 residents) showed a rapid reduction in the prevalence of active yaws infection from 1.8% before mass treatment with azithromycin (30 mg/kg) to a minimum of 0.1% at 18 months, with 84% coverage [9,34]. However, with longer follow-up up to 42 months, a significant increase in cases to 0.4% was observed, indicating that a single round of drug administration may not be enough. These cases were mainly found in individuals who had not received the mass treatment or as new infection in residents. This finding suggests the need for repeated intervention to achieve elimination of yaws. In Ghana, a similar study to assess the effectiveness of this MDA strategy in endemic communities surrounded by other yaws-endemic communities is on-going, i.e., non-isolated communities in contrast to the work in Papua New Guinea. In the pilot study report, the prevalence of active and latent yaws in children reduced from 10.9% before mass treatment to 2.2% at 12 months, in 16,287 children with 89% coverage [7]. The best dosage regimen is also under investigation. An additional benefit would be that azithromycin MDA is also active against trachoma allowing for possible integration of the control of these two NTDs [8].

Scabies is also another disease that potentially may be targeted by MDAs, as oral ivermectin, one of the major drugs used in the control of a number of other NTDs, is also highly effective in scabies. Successful mapping and co-administration of mass treatment studies for scabies have been conducted in the Solomon Islands and integrated with existing programs for trachoma and yaws [10,35,36,37]. In Brazil, selective MDA by targeting communities heavily infected with ectoparasites and enteroparasites using ivermectin considerably reduced the prevalence of a range of coexisting parasitic skin infections, including scabies, pediculosis, cutaneous larva migrans and tungiasis [38]. Similarly, the African Program for Onchocerciasis [39] and the Global Programme to Eliminate Lymphatic Filariasis [40] reported a coincident effect in reducing the number of cases of scabies, an off-target disease. Further integrated activities are planned as part of the assessment of the impact of triple-drug treatment (ivermectin, albendazole, and diethylcarbamazine citrate) for lymphatic filariasis [41]. Use of ivermectin could increase community acceptance of the MDAs through the treatment of skin conditions, particularly if it benefits those severely affected by itching and sores.

Two countries with extensive experience in NTD control, Ethiopia and Fiji are currently developing comprehensive scabies control programs [42]. To further plan on the potential of MDAs for scabies, more studies on dosage and issues related to ivermectin use during pregnancy and in small children are needed. Furthermore, prevalence of scabies is likely to rise again after a certain period of time, and therefore, more operational research is needed to define the best intervals between rounds of MDA. The development of moxidectin, a macrocyclic lactone similar to ivermectin, but with a much longer half-life, for the treatment of human scabies may promote MDAs for scabies [43,44]. Recently in June 2018, the United States Food and Drug Administration (FDA) approved the use of moxidectin for onchocerciasis in patients aged 12 years and older [45].

Although not on the same massive scale as yaws or scabies, there have been attempts to prevent occurrence and interrupt transmission of leprosy using one-dose rifampicin chemoprophylaxis in contact cases (post-exposure prophylaxis). This intervention was tested in Bangladesh in a cluster-randomized controlled trial including 21,711 participants, with successful outcomes at 2-years with a 56.5% reduction in new cases in the intervention group [46]. This protective effect was seen only in the first two years. No additional protective effect was observed after 4 and 6 years, but the total impact of the intervention was still statistically significant after 6 years [47]. A similar result was reported from another study in Indonesia [48]. With the long latent period of leprosy—which can sometimes be more than several decades, long-term follow up is necessary to assess the true effect of interventions for this disease. In the past, chemoprophylaxis (1 to 2 doses of a combination of rifampicin, ofloxacin, and minocycline) has been tested in three Pacific Islands, namely Kiribati, the Federated States of Micronesia, and the Republic of the Marshall Islands, all small islands with a high level of leprosy endemicity [19,20,21]. Now two decades after this intervention, they remain among the countries with the highest new case detection rates for leprosy per population globally [49]. Short-term reduction in new cases of leprosy was observed during the first few years after the intervention, in which active case detection at baseline could have played a considerable role in enhancing the effect of treating some sub-clinical cases. In other words, chemoprophylaxis alone may not be effective unless it is coupled with well-planned active case finding, and the prophylaxis is delivered in the form of several rounds of medication at adequate intervals [26,50]. Currently, the Leprosy Post-Exposure Prophylaxis [51] study—a joint study between India, Indonesia, Myanmar, Nepal, Sri Lanka, and Tanzania using single dose rifampicin—was designed to accelerate the uptake of the evidence for post-exposure prophylaxis and introduce it into national leprosy programs [51]. Recently published interim analysis show that the program has enrolled 5941 index patients and identified a total of 123,331 contacts [52]. Efficacy results are awaited. 

MDAs and prophylaxis may be effective in the control of several skin NTDs, but it is important to note that repeated dosing and other surveillance activities are often needed, as mentioned above. We should also be careful in implementing MDAs/prophylaxis as some drugs may not be safe for use in children, in pregnant women, and in individuals for whom they are contra-indicated. Monitoring for adverse reactions should be carefully done as their occurrence might have a negative impact on the campaigns. How to address these constraints should be elucidated before implementing the strategy on a larger scale. Furthermore, the funds necessary for implementation are not available at the moment, as there is limited interest from donors. Identification of the target populations is also very important not only for the success of the intervention, but also for minimizing cost.

*Key points in implementing mass drug administration:*

1. Careful identification of target populations or case definition
2. Identifying intervals between rounds and number of rounds; some diseases have long latent periods which may be difficult to assess
3. Obtaining strong, coordinated public-private partnerships, including pharmaceutical companies
4. Assessment of secondary effects on other diseases (e.g., leprosy vs. tuberculosis), including increasing the risk of drug resistance
5. Addressing the issues of stigma and discrimination if implementing for contact cases.

## 4. Current Status of Diagnosis and Treatment for Skin NTDs

Few skin NTDs can be confirmed with reliable point-of-care diagnostic tests, and therefore a clinical examination remains the cornerstone of diagnosis. This poses a major challenge, not just in case management of individual patients with skin NTDs, but also in understanding the true epidemiology and disease burden in endemic areas.

To take Buruli ulcer as an example, a study in Cameroon has shown that only 27% among the 327 patients with ulcerative lesions suspected as Buruli ulcer seen at one of their tertiary hospital were confirmed to be Buruli ulcer [53]. Other diagnosis ranged from vascular, bacterial infections, post-traumatic, fistulated osteomyelitis, as well as neoplasia. The team used a combination of Ziehl-Neelsen staining, PCR, and culture from either swab or fine-needle aspiration, skin biopsies, and several systemic tests for diagnostic confirmation [53]. In field settings, these tests are not easy to perform, and it is not difficult to imagine that some patients who have been given a diagnosis based solely on clinical appearance are treated unnecessarily or given the wrong drugs.

PCR targeting IS2404 is currently the test most used for confirming Buruli ulcer. However, access to PCR in many places in West Africa where Buruli ulcer is endemic is very limited. Moreover, adequate skills in sample taking and ensuring transportation of samples in good condition adds to the challenge. It sometimes may take weeks and months to reach the laboratory. It is very difficult to rule out the possibility of false negatives under such conditions. It is also noteworthy that some of the PCR positives with IS2404 include other mycobacterial diseases, for example, *M. marinum*, *M. chelonae*, and *M. smegmatis*, whose distribution in these countries is not yet well understood [54]. Some promising studies are underway in order to develop a rapid diagnostic tool for Buruli ulcer, including the loop-mediated isothermal amplification (LAMP) test [55,56], thin layer chromatography for the detection of mycolactone (lipid toxin produced by the causing bacteria of Buruli ulcer) [57], and antigen detection assays [58].

Table 1 provides the list of diagnostic tests and Table 2 provides treatments currently used for each skin NTD. As treatment of many skin NTDs is of long duration, it is important that diagnostic confirmation be made before initiating treatment. Furthermore, some skin NTDs are associated with considerable stigma and discrimination. Labeling a patient with a specific diagnosis unnecessarily is also another issue that needs to be addressed, and development of easier and more reliable diagnostic methods would also improve these important social aspects associated with skin NTDs. Recently, a point-of-care test for syphilis has been used for early detection of yaws, as they are both from the spirochete bacterium group, replacing the traditional laboratory methods such as rapid plasma reagin (RPR) and *Treponema pallidum* hemagglutination assay (TPHA) [59]. However, a positive test does not necessarily mean that the lesion is due to active yaws, as there are also other pathogenic species in this same group [59]. Further studies for the development of diagnostic tools and reliability checking are needed for all skin NTDs.

Treatments used for skin NTDs are also very limited and many are old drugs or combinations [60]. Integrated advocacy among the skin NTDs may aid in the development of better diagnostic tools and treatment options.

*Key points in diagnosis and treatment of skin NTDs:*

1. Training of local healthcare workers on clinical diagnosis
2. Training of local healthcare workers on diagnostic tests, including sample taking; make a routine for performing diagnostic tests
3. Need for the development of new point-of-care diagnostic tools
4. Developments that enhance laboratory confirmation
5. Need for further investigation of new drugs and regimens for skin NTDs.

## 5. Wound and Lymphedema Management–Cross-Cutting Treatment

**Figure 2:**
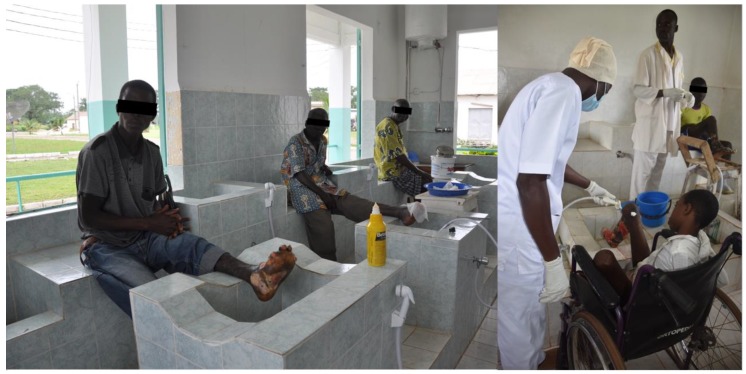
Wound care facility in Côte d’Ivoire for leprosy, Buruli ulcer, and other ulcers.

Despite the availability of different drugs for systemic treatment, the cross-cutting component in the management of a number of skin NTDs is wound (leprosy, Buruli ulcer, yaws, cutaneous leishmaniasis, tropical ulcers, etc.) and lymphedema (lymphatic filariasis and podoconiosis) management, which can be delivered potentially with the same knowledge, skills, and in the same settings. Integration of wound and lymphedema management for skin NTDs is already happening in the field. In many of the co-endemic areas, leprosy and Buruli ulcers are managed in the same facilities, as well as lymphatic filariasis and podoconiosis (Figure 2).

Wounds are among the most frequently encountered skin problems in rural settings in low- and middle-income countries, where skin NTDs are also endemic [53,61]. The causes can range from trauma, burns, bacterial infections as well as to non-communicable diseases such as diabetes and peripheral arterial diseases [53,61]. A large proportion of wounds are at risk of progressing to a chronic stage when not supported by proper diagnosis and wound care. Skills in evaluating the abnormal signs and symptoms, such as when to stop using topical antiseptics, when to suspect secondary infection, when to suspect malignant alteration, are skills that are lacking as there is limited training and the need for the expertise in wound care is not well recognized. A study conducted in Ghana and in Benin interviewing health care personnel dealing with the wound management of Buruli ulcer patients reported that standard of wound care differed greatly both between personnel and between institutions [62]. Limited accessibility to clean water can lead to prolonged secondary infection, as well as infrequent dressing changes due to poor availability of dressing materials and access to health facilities. Use of traditional medicines, and sometimes use of local folk treatments such as ash and toothpaste, hinder the normal wound healing process.

The most important component in wound management is achieving a clean wound bed with red granulation tissue, protected from infection and trauma [63,64]. Securing clean water to wash the wound surface regularly and thoroughly is the foremost priority; having normal saline solution is ideal, if not, tap water fit for drinking or cooled boiled water can be used for this purpose [65,66] (Figure 2). There is currently a wide range of wound dressing products designed for use in developed countries to keep the wound bed moist. They are often costly, although they may be useful if they shorten the wound healing time [67,68,69,70]. Nonetheless, there are good wound care techniques applicable in places where patients with ulcerative skin NTDs reside, using materials that are readily available, e.g., saline, vaseline [65]. The use of honey should not be dismissed although further assessment is needed [71]. Removal of necrotic tissue on the wound surface is another important component in wound management [63,64], which can be achieved through training of the local healthcare providers. 

The main pillar in management of lymphedema is also based on hygiene—regular and thorough washing with soap and water—and skin care. A systematic review by Stocks et al., reported that participation in hygiene-based lymphedema management decreased the incidence of acute dermatolymphagioadenitis (ADLA) by one-third [72]. These inflammatory episodes, which are characterized by pain, fever, and swelling of the affected limb create a vicious cycle as they further erode lymphatic function stimulating more fibrosis and it is therefore important to prevent these [73]. Protecting the skin barrier function with simple emollients such as vaseline as well as limb exercise are also key to reducing inflammation and swelling [74,75].

Studies investigating wound or lymphedema care in resource-limited settings are lacking. The severe impact a wound or a lymphedema can have on the quality of life of those affected is under-recognized [76,77,78,79,80,81,82]. Furthermore, findings from some cost analyses carried out in the developed world show a strikingly high cost burden [83,84], and this also needs to be assessed in resource-limited settings. There has been a recent study investigating the cost of wound care for cutaneous leishmaniasis in Afghanistan comparing different methods [85]. More studies of this kind will further improve the efficient management of skin NTDs. There are also other opportunities for integration between skin NTDs and chronic diseases, such as diabetes, in limb care or between other interventions such as clean water, better sanitation and hygiene (WASH) [77], which should be further explored.

*Key points in wound and lymphedema management:*

1. Implementation of a simple algorithm utilizing inexpensive and easily obtainable products for wound management/lymphedema management
2. Better use of those resources that are available in the local setting
3. Cost-analysis
4. Training and deployment of helpers including both local health care workers and “the expert patient”.

## 6. Self-Morbidity Management to Improve Outcomes and Social Inclusion

Some skin NTDs, when diagnosed late, can lead to life-long disabilities and disfigurement. This may result from diseases with extensive ulceration or lymphedema as listed in the above section (Buruli ulcer, leprosy, tropical ulcers, lymphatic filariasis, podoconiosis, etc.) but there are other causes. For instance, ulceration, disabilities and deformities in a leprosy patient can also occur as a result of peripheral nerve damage—clawing toes, drop hands and feet, etc. Often patients with limb lesions from mycetoma are managed by amputation [80] as is the case with other severe forms of ulceration. Early identification and treatment can reduce this.

As outlined in the above section, the use of locally available methods and materials at low cost, which are also culturally accepted, are key in achieving a sustainable morbidity management program. A good example of this is a study by Narahari et al. in India, where the group developed an integrative treatment protocol for morbidity reduction of lymphatic filariasis by combining Ayurvedic exercises, compression therapy and modern dermatology drugs to treat bacterial entry points [77]. While the treatments for such conditions may include complex procedures such as lymphovascular shunts and debulking surgery, this integrative and non-invasive treatment, widely available in the local Indian settings was strikingly successful in reducing the volume of the limbs and also led to fewer episodes of ADLA. 

In Ethiopia, integrated morbidity management for lymphatic filariasis and podoconiosis are in the process of implementation by the health ministry and partners, with some highly successful outcomes. Integration for leprosy and Buruli ulcer are already happening in the field, even in places where the national public health programs are still organized in a vertical pattern. As leprosy and Buruli ulcer share the same goal, which is prevention of disability (POD), the two diseases might be expected to benefit from synergies in management. It is of note that they also share similarities in diagnosis and treatment as the causative organisms come from the same mycobacterial disease group, and advocacy strategies have focused on more integration between the two [86]. 

**Figure 3:**
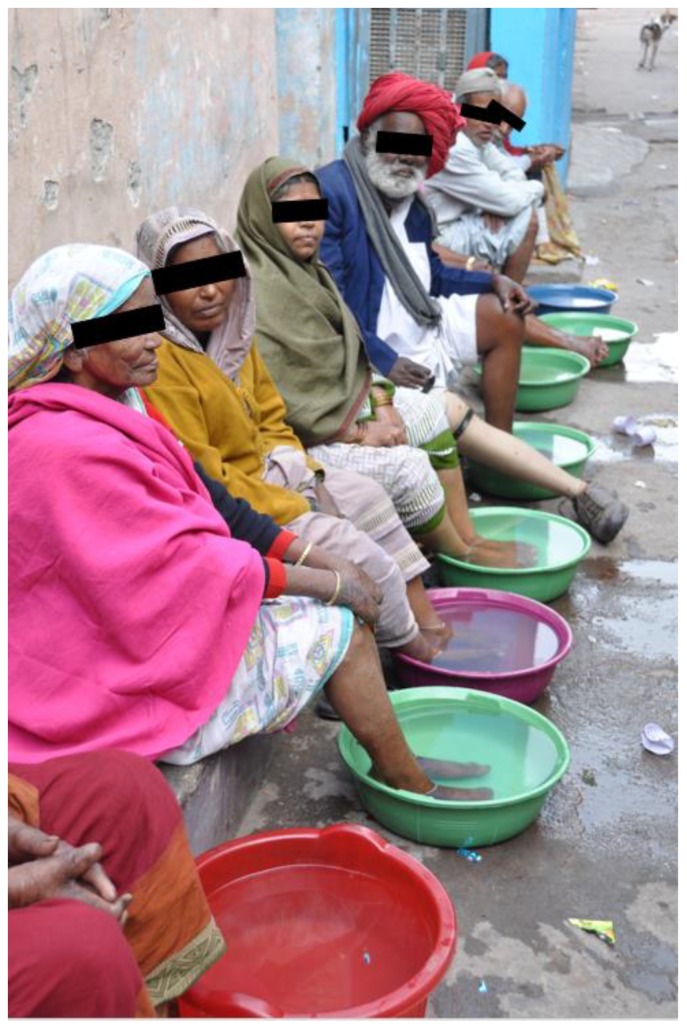
Education on self-skin care for prevention of disabilities (POD) for people affected by leprosy in a village in India.

As these skin NTDs are chronic, management of individual patients, including skin care and dressing or compression at home are critical in achieving success in treatment outcomes. A systematic review by Douglass et al., reported that intensity of training of patients in self-care practices and frequency of monitoring improved treatment outcomes [87], while similar findings were reported by Sathiaraj et al. for leprosy [88] (Figure 3).

Better treatment outcomes can be achieved when there is involvement of patients themselves and their carers. Self-care and carer assisted programs are also important in helping to reduce the work burden of local healthcare providers, as without these integrative techniques may even add to their work load. The success of Narahari et al. in their morbidity management for lymphatic filariasis was possible through recruiting patients accompanied by a family member willing to support self-care at home [77]. Carers must be willing and actively take part in the management of the family member, including support in dressing changes and skin care, relief of odor and pain, nourishment, helping with mobility, encouraging them to maintain adequate hygiene by bathing and, above all, making sure that they feel welcome in their society so that they can live full lives, marry, and achieve employment. [89]. As emphasized here, social inclusion is indeed another critical dimension in case management of skin NTDs, which can benefit from a synergized approach to skin NTD integration.

*Key points in self-morbidity management to improve outcomes and social inclusion:*

1. Use of locally available methods and materials at low cost
2. Patient training for self-care
3. Patient empowerment
4. Carer training
5. Interventions to promote social inclusion.

## 7. Training and Referrals

No disease control programs can be successful without good training of the local healthcare providers and workers, who are the closest to patients. This is certainly true for skin NTDs as clinical diagnosis remains the most readily available diagnostic measure and an important entry point for disease control of most skin NTDs. Most of the successful projects introduced here have been achieved through good and effective training. One of the major strategies deployed in the Ethiopian lymphatic filariasis-podoconiosis integrated program has been training, guideline development featuring a simple algorithm on clinical assessment, treatment and referral needs, and a defined care package [90]. This initiative also developed and rolled out a teaching video for healthcare workers on integrated morbidity management.

For integration, it is essential to provide suitable training for local healthcare providers on skin NTDs, as well as common skin diseases as these are more frequent and a better knowledge of these diseases will empower them. A well-trained healthcare provider who gains the confidence and acceptance of the population is key to achieving good results. In a study in Côte d’Ivoire during which we conducted screening of schoolchildren with skin NTDs, training was given with this consideration and it was effective in extending the program to a larger population with skilled local healthcare providers [12]. These healthcare providers perceived that the training was very helpful for their daily practices and their involvement in our project has been continuous. WHO and the expert panel group recently developed a training manual “Recognizing neglected tropical diseases through changes on the skin: a training guide for front-line health workers”, which was developed with the vision to support such activities [91]. As the endemicity of diseases varies from one place to another, the document can be modified to be country/context-specific.

Along with capacity building at field level, clear referral pathways, e.g., for clinical consultation, referral for hospitalization, sending of samples to laboratories, etc. need to be established or strengthened to ensure better integrative management of skin NTDs, irrespective of disease type. We need to bear in mind that there is a wide range of skin diseases, other than skin NTDs, in which diagnosis are difficult, and some can even be fatal, e.g., acute infections, drug eruptions, and cutaneous malignancies. A study in Brazil where leprosy services have been decentralized has demonstrated the importance of referral centers in support of local health services in treating skin diseases [92].

With increasing availability and accessibility to mobile phones and internet, teledermatology is one way forward for establishing an adequate training, support and referral system. In a comprehensive project for mycetoma management in Sudan, a computer application on computer tablets or smartphones connected house-to-house survey medical teams, regional tertiary center, and experts at national level [80]. In Malawi and Ghana, a community-led SMS reporting tool for the rapid assessment of lymphatic filariasis morbidity burden was successful in involving community-based health surveillance workers/volunteers to participate in reporting and continuous monitoring of patients [93]. A similar approach could be established for other skin NTDs with respect to co-endemicity, and involvement of dermatologists will enable the approach. However, as there is a shortage of dermatologists in these settings, besides mobilization of local dermatologists, establishment of networks extending beyond the country may be necessary [94]. Two examples of such projects are described in this issue [95,96].

## 8. Next Steps

Integration of programs for skin NTDs is happening at national level. Some of the countries that have integrated their national public health programs for leprosy and Buruli ulcer include Benin, Cameroon, Congo Brazzaville, Gabon, Papua New Guinea and Togo; Nigeria combines leprosy, Buruli ulcer, and tuberculosis (TB). Some countries such as Côte d’Ivoire, Democratic Republic of Congo, and Ghana [86] still have separate control programs. However, these countries are also exploring integration of these skin NTDs, including yaws. Identifying the most appropriate combination of diseases, based on disease control measures and co-endemicity, is the key to successful integration.

Identifying opportunities beyond NTDs, such as integration with TB programs (DOTS), WASH, and non-communicable diseases (NCDs) may open a new door. Recent screening activities in Kiribati for NCDs and TB produced off-target results—identification of new leprosy cases. As the underlying risk factors for skin NTDs are poverty and poor hygiene, integration with poverty reduction programs should also be explored. A systematic review by Tomczyk et al. reported that access to footwear use significantly reduced the incidence of Buruli ulcer, cutaneous larva migrans, tungiasis, hookworm infection, soil-transmitted helminth infection, strongyloidiasis, and leptospirosis [97].

Whatever the case, attention needs to be paid to facilitating a reduction in the workload of front-line healthcare workers. Consideration is also needed as to how well any program can retain workers with skills needed in management of skin NTDs, to improve early detection and treatment outcomes, prevent or minimize disabilities, and ease the burden on governments and NGOs [86].

Establishment of expert working groups for guideline making, training materials, advocacy, etc. could facilitate this process. Recently, WHO and the working group on leprosy and yaws published guidelines for each disease [59,98]. An international alliance for the control of scabies (IACS) has been formed to advance collaboration [42,99]. Further research in the field demonstrating the burden of disease, and the effectiveness and synergies of control strategies, may increase the visibility of skin NTDs within the global health agenda and attract greater attention from international agencies and donors.

## 9. Conclusions

Many skin NTDs disproportionately affect the world’s most disadvantaged people living in the low- and middle-income countries. They are often chronic in nature and may lead to chronic secondary conditions, including life-long disabilities and disfigurement, creating a vicious cycle for individuals, families, and society. Until recently, they were the most neglected among other NTDs as their disease control measures rely mostly on individual case management and any visible impact in the control achievements was limited. The present drive towards integration of skin NTDs together or with other programs has significant potential to reverse this limitation. There is a body of existing practice and field research for each disease among those interested in skin NTDs, which have the potential to further enhance the process of integration. There has also been new research on skin NTDs including approaches to integration that have had encouraging outcomes. It is essential that we build on these good practices and the lessons learned in order to formulate sound strategies for the shared goal of the fight against NTDs.

## Figures and Tables

**Table 1:** Diagnostic methods and tools for skin NTDs.

	Pathogen	Rapid Diagnostic Test	PCR	Microscopy	Culture	Serology	Others
**Buruli ulcer**	*Mycobacterium ulcerans*	X	O	O	O	X	LAMP test, thin layer chromatography, antigen detection assays under development
**Cutaneous leishmaniasis (CL)/mucocutaneous leishmaniasis (ML)**	*Leishmania* species	X	O	O Skin smears	O	X	LAMP test, antigen detection assays under development (Montenegro skin test)
**Lymphatic filariasis (LF)**	Microfilaria (*Wuchereria bancrofti*, *Brugia malayi*, etc.)	O	O	O Blood smears	X	∆ Anti-filarial antibodies	Ultrasonography
**Onchocerciasis**	Microfilaria (*Onchocerca volvulus)*	O	O	O Skin snips	X	∆ Anti-filarial antibodies	Direct observation of adult worms from nodule(s), slit-lamp eye exam, serological and antigen tests under development
**Leprosy**	*Mycobacterium leprae*	X	O	O	X	∆ Anti-PGL-I antibody	Thickened nerves, loss of muscle strength, anesthetic skin lesion
**Mycetoma**	Fungal or bacterial species	X	O	∆	O	X	X-rays, CT, ultrasonography, etc.
**Podoconiosis**	Irritant alkalic clay soils	N/A	N/A	N/A	N/A	N/A	Location, history, clinical findings; negative results for LF and other lymphedema-causing diseases; genetic susceptibility
**Scabies**	*Sarcoptes scabiei* var. *hominis*	X	∆	O	X	X	Dermatoscopy, burrow ink test
**Tungiasis**	*Tunga penetrans* (sand fleas)	X	∆	O	X	X	Direct observation of adult fleas and eggs from skin lesion(s), dermatoscopy
**Yaws**	*Treponema pallidum* subsp. *pertenue*	O	O	O	O	PRP, TPHA, FTA-ABS, etc.	Diagnostics for differentiation of *Treponema pallidum* species under development

O = available; ∆ = available but not confirmatory or standardized; X = unavailable.

**Table 2:** Treatment and management for skin NTDs.

	Medical Treatment	Surgery	Wound or Lymphedema Management	Self-Morbidity Management	Prevention
**Buruli ulcer**	**Standard:** Oral rifampicin + clarithromycin for 8 weeks **Other tested regimens:** Oral rifampicin + either 1 or 2 of [ciprofloxacin, ethambutol, mofloxacin, amikacin, etc.]	Yes	Yes	Yes	Limited, route of transmission unknown (Stay away from contaminated water sources)
**Cutaneous leishmaniasis (CL)/mucocutaneous leishmaniasis (ML)**	Individualized treatment depending on species (no standard) Amphotericin B deoxycholate, pentavalent antimonials, fluconazole, ketoconazole, miltefosine, paromomycin ointment, etc. **Simple CL** lesion(s) with low ML-risk: natural healing may occur **Complex CL** lesion(s) with high-ML risk, severe lesion(s), immunocompromised persons, etc.: treat all cases	No	Yes	No	Limited (Avoid sand fly bites)
**Lymphatic filariasis (LF)**	Oral albendazole ± [diethylcarbamazine (DEC) or ivermectin] When long-term treatment is possible: Oral DEC (1–12 days) ± doxycycline for 4 to 6 weeks Note: DEC contraindicated in onchocerciasis endemic sites	Yes	Yes	Yes	Avoid mosquito bites, MDAs, vector control, etc.
**Onchocerciasis**	Oral ivermectin	Yes	No	No	Avoid blackfly bites, MDAs, vector control, etc.
**Leprosy**	**Multiple drug therapy (MDT):** Oral rifampicin + dapsone + clofazimine for 6 to 12 months	Yes	Yes	Yes	Contact tracing and early detection; prophylaxis with one-dose rifampicin in trial
**Mycetoma**	Antibiotics or antifungals depending on species for long-term	Yes	Yes	Yes	Footwear
**Podoconiosis**	N/A	Yes	Yes	Yes	Footwear
**Scabies**	Oral ivermectin, 1–2 doses 1 week apart	No	No	No	Early diagnosis and treatment of contacts, possible MDAs in endemic communities
**Tungiasis**	None (primary treatment: hygienic mechanical removal of fleas), antibiotics if secondary infection is indicated	No	Yes	No	Footwear
**Yaws**	Single oral azithromycin or injectable benzathine penicillin	No	Yes	No	Contact tracing and early detection, possible MDAs in endemic communities
